# Iodine Deficiency and Excess Coexist in China and Induce Thyroid Dysfunction and Disease: A Cross-Sectional Study

**DOI:** 10.1371/journal.pone.0111937

**Published:** 2014-11-06

**Authors:** Yang Du, Yanhui Gao, Fangang Meng, Shoujun Liu, Zhipeng Fan, Junhua Wu, Dianjun Sun

**Affiliations:** Center for Endemic Disease Control, Chinese Center for Disease Control and Prevention, Harbin Medical University; Key Lab of Etiology and Epidemiology, Education Bureau of Heilongjiang Province & Ministry of Health (23618504), Harbin, China; Rutgers University, United States of America

## Abstract

**Background:**

In spite of the salt iodization, iodine deficiency disorders (IDD) have not been sustainably eliminated in China. There are coastal areas with low iodized salt coverage rates (iodine nutrition is inadequate) and other areas with excessive amounts of iodine in the drinking water.

**Objective:**

This study aimed to clarify the association of iodine deficiencies resulting from a low coverage rate of iodized salt, excess iodine intake from drinking water with thyroid function and disease in adults.

**Design:**

A cross-sectional study was conducted in adults in different iodine nutrition areas in three provinces in China.

**Results:**

The prevalence of thyroid nodules was 15.52%, 8.66% and 22.17% in the iodine excess, sufficient and deficient groups, respectively. The prevalence of subclinical hypothyroidism was 20.09%, 10.41%, and 2.25% in the excess, sufficient and deficient iodine groups, respectively. The prevalence of subclinical hyperthyroidism and overt hyperthyroidism in the iodine deficient group was higher than that in the iodine excess group (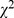 = 9.302, *p* = 0.002) and iodine sufficient group (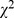 = 7.553, *p* = 0.006). Thyroid-stimulating hormone (TSH) was significantly correlated with excess iodine intake (*β* = 1.764,*P* = 0.001) and deficient iodine intake (*β* = −1.219, *P* = 0.028).

**Conclusions:**

Thyroid nodules are more likely to be present in the iodine excess and deficient areas than in the iodine sufficient areas. Subclinical hyperthyroidism and overt hyperthyroidism are more likely to be prevalent in the iodine deficient areas than in the iodine excess or sufficient areas. Subclinical hypothyroidism is more likely to be prevalent in the high iodine intake areas than in the iodine deficient or sufficient areas. Median TSH may be deemed as an alternative indicator for monitoring the iodine nutrition status of the adult population in iodine excess and deficient areas.

## Introduction

Iodine is an essential trace element required for the synthesis of thyroid hormones (including thyroxine and triiodothyronine), and it is indispensable for normal growth and fetal brain development in human beings [1], [2]. Both iodine deficiency and excess intake may lead to thyroid dysfunction or disease. Iodine deficiency has widespread implications, which include cretinism, endemic goiter, intellectual impairments, increased pregnancy loss and infant mortality [3], [4], [5], [6], [7]. Excess iodine intake may lead to endemic goiter and thyroid dysfunction [8], [9]. It is reported that thirty countries remain iodine deficient and ten countries have excessive iodine intake across the world [10]. The best strategy to prevent iodine deficiency disorders (IDD) is the addition of iodine to dietary salt in most countries [11]. To date, IDD has been primarily controlled in China. A common reason for iodine excess intake in many countries is the over iodization of salt and/or poor monitoring of salt iodization [12]. However, unlike other iodine excess countries, China has excess iodine areas resulting from high iodine concentrations in water [13]. Moreover, there are iodine deficient areas due to the low coverage rate of iodized table salt along the coast. Previous studies of iodine excess or deficiency have primarily focused on the vulnerable populations such as children, pregnant women and lactating women [14], [15], [16], [17], [18]. The last study in China was performed in Henan, Hebei, Shanxi and Shandong province (four main excess iodine areas by historical Yellow River flushing, and are representative of Chinese iodine excess area) and published in 2014, this study focused on the iodine excess in the vulnerable population, and suggested that excessive iodine intake was associated with impaired thyroid function of children, pregnant women and lactating women [19]. Studies on the impact of iodine deficiency or excess on the adults are scarce. Therefore, the main objective of this study is to clarify the relationship between adult iodine malnutrition (including iodine deficiency due to low coverage rate of iodized salt and excess iodine intake from drinking water) and thyroid abnormalities (dysfunction and disease).

## Subjects and Methods

### Subjects

According to the recent national IDD surveillance data and water iodine surveillance data, three populations with different iodine nutrition levels were selected in three provinces. Fengrun and Yangfang village in Qingxu County, Dongwenzhuang village in Xiaodian District of Shanxi Province, and Caizhuang village in Cao County of Shandong Province were selected as areas where residents consume high iodine water. Shuangzhai, Houhuangtai, Lizhuang, Anrong and Hutong villages in Shanyin County and Xicuan village in Xiaodian District of Shanxi Province were selected as areas where residents have sufficient iodine nutrition. Tieshangang District in Beihai City, Guangxi Autonomous Region, was selected as a coastal area with low coverage rates of iodized salt and regarded as an area with iodine deficiency. The income of most participants in these areas is similar (4000–5000 RMB yuan/person/year, equivalent to USD 640–800/person/year). A cluster random sampling method was adopted in these areas. All participants who had lived in the region for more than 10 years and were older than 18 years were eligible for recruitment. Subjects taking anti-thyroid drugs during the time of this study were excluded.

### Methods

#### Questionnaire

A standard questionnaire was designed to obtain demographic information, including name, gender, age, personal or family history of thyroid disease, smoking and drinking habits, economic income and source of drinking water and drinking duration. For the smoking habit, participants were divided into four grades: non smoker, mild smokers (1–10 cigarettes/day), moderate smokers (11–20 cigarettes/day) and heavy smokers (>20 cigarettes/day). For the alcohol consumption habit, participants were divided into three categories: non-drinking, occasional light consumption (<1 time/week) and heavy consumption (≥1 times/week). The investigation was performed face to face in dialect by well-trained staff.

#### Drinking Water Collection and Analysis

In the preselected high iodine water and iodine sufficient villages, at least four water samples were collected from the east, west, south, north and central directions of each village with scattered water supplies; one water sample was collected from each village with centralized water supplies. In the preselected iodine deficient area (Tieshangang District), each participant provided a water sample. Each water sample was more than 15 ml and kept at 4°C until analysis. The iodine concentration of the drinking-water was determined by arsenic–cerium catalytic spectrophotometry [20].

#### Urine Sample Collection and Analysis

Morning spot urine samples were collected in clean plastic tubes for each participant. Urinary iodine concentration was determined by the acid digestion method (As^3+^−Ce^4+^catalytic spectrophotometry [21]). The internal quality control samples for urine iodine and water iodine were provided by the China National Iodine Deficiency Disorders Reference Laboratory.

#### Coverage Rate of Iodized Salt

In the preselected iodine excess or sufficient areas, the iodized salt coverage rates were calculated by the Information Management System of National Iodized Salt Surveillance, which is managed by our organization. In the preselected iodine deficient area (Tieshangang District), each participant provided a table salt sample of at least 100 g. The salt iodine levels were determined using the General Test Method of the salt industry for the determination of iodine [22]. The standard for salt iodine level in Shandong, Shanxi province and Guangxi Autonomous Region is 25 mg/kg. The salt type was defined as follows: non-iodized salt, salt iodine less than 5 mg/kg, qualified iodized salt, salt iodine more than 18 mg/kg and less than 50 mg/kg, others, unqualified iodized salt.

#### Blood Sample and Analysis

Blood samples were collected from the cubital vein and stored in a glass tube. The serum samples were prepared by centrifugation (3000 r/m, 10 mins) after standing for an hour and were frozen at −70°C until determination. Serum thyroid-stimulating hormone (TSH), free triiodothyronine (FT3), free tetraiodothyronine (FT4), thyroid peroxidase antibody (TPOAb) and thyroglobulin antibody (TGAb) were tested. These indexes were tested by chemiluminescent immunoassay (Fluorometric magnetic beads enzyme immunoassay, TOSOH Corporation, Japan). The thyrotropin receptor antibody (TRAb) was tested by chemiluminescent immunoassay (Snibe Co., Ltd, China).

#### Thyroid Ultrasound

Thyroid ultrasonography was performed using a 7.5-MHz transducer by one experienced examiner (mainly measure the thyroid volume, nodule diameter and echo). The thyroid lobe volume was calculated by measuring the depth (d), the width (w) and the length (l) of each lobe by the following formula: V (ml)  = 0.479×d×w×1 (mm)/1000. The thyroid volume was recorded as the sum of both lobes. The normal volume for adult males and females is less than 25 ml or 18 ml, respectively [23].

#### Diagnostic Criteria for Thyroid Diseases

The diagnostic criteria for thyroid diseases were listed in Table 1 [24], [25].

**Table 1:** Diagnostic criteria for the thyroid diseases included in this study.

Thyroid disease	Diagnostic criteria*
Goiter	Thyroid volume >25 ml (male) or >18 ml (female)
Diffuse	Goiter without nodules
Nodular	Goiter with nodules >10 mm in diameter
Single nodule	Normal thyroid volume with a single nodule >3 mm in diameter
Multiple nodules	Normal thyroid volume with >2 nodules >3 mm in diameter
Overt hypothyroidism	TSH>5.57 mIU/L, FT4<10.06 pmol/L
Subclinical hypothyroidism	TSH>5.57 mIU/L, FT4 within the normal range
Over hyperthyroidism	TSH<0.38 mIU/L; FT4>23.99 pmol/L, FT3>5.85 pmol/L, or both
Subclinical hyperthyroidism	TSH<0.38 mIU/L, FT3 and FT4 within the normal ranges
Graves' disease	Hyperthyroidism. Diffuse goiter on ultrasound. TPOAb>2.6 IU/ml or TRAb>30 IU/ml
Autoimmune thyroiditis	Hypothyroidism or subclinical hypothyroidism. TPOAb>2.6 IU/ml or TgAb>14.58 IU/ml
Hashimoto's thyroiditis	With goiter
Atrophic thyroiditis	Without goiter

^*^Reference ranges: FT3, 2.77–5.85 pmol/L; FT4, 10.06–23.99 pmol/L; TSH, 0.38–5.57 mIU/L; TPOAb, 0–2.6 IU/ml; TgAb, 0–14.58 IU/ml; TRAb, 0.11–30 IU/ml. Reference ranges were provided by the second affiliated hospital of Harbin Medical University.

#### Statistical Analysis

All data were recorded with EpiData3.1, the data processing and statistical analyses were performed using IBM SPSS statistics version 19 and SigmaPlot 12.5 software. Comparison of the proportions among the three groups was performed by the chi-square test (α = 0.05); if the null hypothesis was rejected, pairwise comparisons were performed (α = 0.0125). Normally distributed data were expressed as the mean ±SD; abnormally distributed data were described as the median with the 25th and 75th percentiles. Binary logistic regression analysis was used to calculate the odds ratio (OR) and 95% Confidence Intervals (CIs) for thyroid diseases by iodine nutrition status and other risk factors. The dependent variables entered into the logistic regression models were goiter, autoimmune thyroiditis, subclinical hyperthyroidism and overt hyperthyroidism, nodules and subclinical hypothyroidism. Gender, age, smoking status, alcohol consumption, goiter, nodules, iodine status, TPOAb positive and TGAb positive were entered as explanatory variables. First, univariate logistic regression was performed to select the potentially significant variables for the multivariate model. Then, based on the results of univariate logistic analysis, the significant variables, gender and age were put into the multivariate logistic model. The pairwise comparisons of the TSH values among the three groups were compared by the Kruskal-Wallis test. The relativity between TSH and urinary iodine concentration was tested using the Spearman rank correlation.

#### Ethical Statement

The study was approved by the Ethical Review Board of Harbin Medical University (HMUIRB20120021). Written informed consent was obtained from all participants ahead of the survey. No specific permits were required for the locations or activities associated with the drinking water sample collection in this field study. The locations were not privately owned or protected in any way and this field study did not involve endangered or protected species.

## Results

A total of 2147 adults were recruited for this survey, the sample sizes were presented in Table 2. The demographic characteristics of the participants were presented in Table 3.

**Table 2:** A summary of the samples collected and the iodine nutrition status of the survey sites.

Survey sites		Participant numbers	Water samples		Urine samples		Blood samples	Ultrasound samples	Table salt samples
			n	Median (Interquartile range) (µg/L)	n	Median (Interquartile range) (µg/L)			
Iodine excess group	Caizhuang	285	246	272.98 (171.95,393.33)	281	473.38 (270.51,758.51)	282	285	—
	Dongwenzhuang	156	1	629.90	145	1183.96 (793.26,1557.11)	156	156	—
	Fengrun	145	1	571.20	108	896.55 (525.03,1264.95)	137	139	—
	Yangfang	344	1	460.20	254	823.90 (573.05, 1219.88)	286	335	—
	Sum	930	249	—	788	750.18 (451.84, 1188.55)	861	915	—
Iodine sufficient group	Xicuan	122	2	77.77	116	264.72 (195.85, 380.04)	122	121	—
	Shuangzhai	68	20	23.15 (9.94, 32.96)	56	272.41 (175.95, 328.72)	56	66	—
	Houhuangtai	163	20	10.90 (1.62, 19.52)	111	188.45 (112.48, 258.75)	146	158	—
	Lizhuang	65	20	13.13 (1.0, 30.92)	53	229.50 (108.39, 288.78)	61	63	—
	Anrong	86	25	7.69 (1.46, 11.37)	71	239.50 (124.10, 324.50)	80	78	—
	Hutong	46	20	15.29 (10.51, 28.02)	43	209.99 (126.94, 293.20)	44	45	—
	Sum	550	107	—	450	228.70 (134.13, 322.73)	509	531	—
Iodine deficient group	Tieshangang	667	336	2.84 (1.93,5.87)	642	62.03 (36.24, 99.10)	667	636	280

**Table 3:** Demographic characteristics of the participants in the three different iodine nutrition groups.

Characteristics		Iodine excess group	Iodine sufficient group	Iodine deficient group
Participants number		930	550	667
Gender				
	Male	281	212	157
	Female	649	338	510
Age				
	Mean ±SD	55.46±12.44	57.71±12.11	51.89±15.10
	≤40	181	91	247
	41–60	432	254	214
	61-	317	205	206
Education level 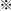				
	Illiteracy	238	91	61
	Primary school	396	282	197
	Junior high school	227	140	217
	Senior high school	55	16	120
	College	14	5	72
Marital status▴				
	Single	4	2	27
	Married	914	533	614
	Divorced/widowed	12	2	26
Smoking★				
	None	789	432	628
	1–10	29	33	21
	11–20	29	3	12
	>20	66	77	6
Alcohol consumption△				
	None	809	490	592
	Light	65	28	51
	Heavy	37	22	24

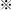
16 missing.

▴ 13 missing.

★ 22 missing.

△ 29 missing.

### Iodine Concentration in Urine Sample

Each village was a unit, the villages were divided into excess, sufficient and deficient groups according to the median urinary iodine (MUI) of each village. The grouping criteria was as follows: iodine excess group, MUI>400 µg/L; iodine sufficient group, 100 µg/L<MUI<300 µg/L; and iodine deficient group, MUI<100 µg/L. The MUI of the participants was 750.18 µg/L, 228.70 µg/L and 62.03 µg/L for iodine excess, sufficient and deficient groups, respectively, as shown in Table 2.

### Iodine Concentration in Drinking Water

The detailed iodine concentrations in the drinking water of each survey site were presented in Table 2. The median iodine concentration of the drinking water ranged from 272.98 µg/L to 629.90 µg/L in the iodine excess group and 7.69 µg/L to 77.77 µg/L in the iodine sufficient group. The median in the iodine deficient group was 2.84 µg/L.

### Coverage Rate of Iodized Table Salt

In Shanyin County, the coverage rates of iodized salt and qualified iodized salt were 99.67% and 98%, respectively. In high iodine water areas of Xiaodian District and Cao County, the coverage rates of iodized table salt were 0% and 1.67%, respectively. In the iodine deficient areas of Tieshangang, the coverage rates of iodized salt and qualified iodized salt were 24.29% and 17.14%, respectively.

### The Prevalence of Thyroid Diseases

#### Nodule

**Figure 1:**
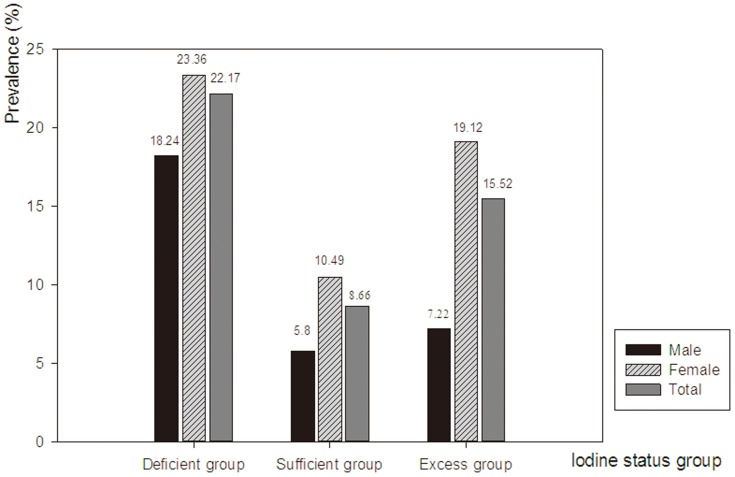
The prevalence of thyroid nodules among different iodine nutrition groups.

The prevalence of thyroid nodules in the different iodine nutrition groups was presented in Table 4. For single and multiple nodules, the prevalence of thyroid nodules differed significantly among the three groups (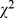 = 39.779, *p* = 0), between the iodine deficient group and iodine sufficient group (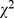 = 39.234, *p* = 0), between the iodine excess group and iodine sufficient group (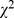 = 13.964, *p* = 0), and between the iodine excess group and iodine deficient group (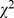 = 11.125, *p* = 0.001). The prevalence of thyroid nodules for males and females was shown in Figure 1. In males, a significant difference in the prevalence of thyroid nodules was found among the three groups (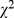 = 18.401, *p* = 0), between the iodine deficient group and iodine sufficient group (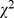 = 13.67, *p* = 0), and between the iodine deficient group and iodine excess group (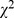 = 11.916, *p* = 0.001), but not between the iodine excess group and iodine sufficient group (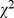 = 0.389, *p* = 0.533). In females, the prevalence of thyroid nodules differed significantly among the three groups (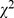 = 21.463, *p* = 0), between the iodine excess group and iodine sufficient group (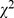 = 11.775, *p* = 0.001), and between the iodine deficient group and iodine sufficient group (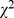 = 21.629, *p* = 0), but not between iodine excess group and iodine deficient group (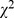 = 2.998, *p* = 0.083). The prevalence of single nodule differed significantly among the three groups (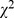 = 20.624, *p* = 0), between the iodine excess group and iodine sufficient group (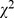 = 10.999, *p* = 0.001), and between the iodine deficient group and iodine sufficient group (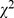 = 20.809, *p* = 0), but not between the iodine excess group and iodine deficient group (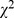 = 2.96, *p* = 0.085). For multiple nodules, the prevalence of nodules in the iodine deficient group was higher than that in other groups (Iodine sufficient group vs. Iodine deficient group, 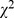 = 17.918, *p* = 0; Iodine excess group vs. Iodine deficient group, 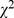 = 12.933, *p* = 0), as shown in Table 4. Logistic-regression analysis indicated that female or elder age, excess iodine intake or iodine deficiency, smoking 11–20 cigarettes per day and goiter were risk factors for developing thyroid nodules (Table 5).

**Table 4:** Prevalence of thyroid nodules and goiter within the different iodine nutrition groups.

Thyroid diseases	Iodine excess group number of cases (percent)	Iodine sufficient group number of cases (percent)	Iodine deficient group number of cases (percent)
Nodules	142 (15.52)	46 (8.66)	141 (22.17)
Single nodule	121 (13.22)	40 (7.53)	104 (16.35)
Multiple nodules	21 (2.30)	6 (1.13)	37 (5.82)
Goiter	23 (2.51)	12 (2.26)	22 (3.46)
Diffuse	21 (2.30)	9 (1.69)	15 (2.36)
Nodular	2 (0.22)	3 (0.56)	7 (1.10)

**Table 5:** Associations between thyroid diseases and different iodine nutrition status and other potential risk factors.

Factors		Sub-group Number	Goiter	Autoimmune thyroiditis	Subclinical hyperthyroidism and overt hyperthyroidism	Nodules	Subclinical hypothyroidism
			OR (95% CI)	OR (95% CI)	OR (95% CI)	OR (95% CI)	OR (95% CI)
Gender	Female	1497	1	1	1	1	1
	Male	650	0.251 (0.097, 0.647)**	0.849 (0.558, 1.292)	0.214 (0.050, 0.909)*	0.478 (0.332, 0.689)**	0.666 (0.473, 0.940)*
Age	≤40	519	1	1	1	1	1
	41–60	900	0.687 (0.319, 1.478)	1.472 (0.856, 2.531)	0.575 (0.280, 1.180)	1.602 (1.146, 2.240)**	1.363 (0.907, 2.046)
	61–	728	1.583 (0.775, 3.233)	1.465 (0.830, 2.588)	0.216 (0.071, 0.653)**	1.913 (1.354, 2.702)**	1.186 (0.768, 1.832)
Iodine status	Sufficient	550				1	
	Excess	930				1.865 (1.299, 2.678)**	
	Deficient	667				2.973 (2.046, 4.321)**	
Iodine status	Deficient	667	1	1	1		1
	Sufficient	550	0.979 (0.448, 2.143)	2.162 (1.168, 4.001)*	0.401 (0.146, 1.102)		5.791 (3.161, 10.611)**
	Excess	930	0.981 (0.524, 1.836)	3.012 (1.742, 5.209)**	0.404 (0.186, 0.880)*		11.717 (6.742, 20.362)**
Smoking	Never	1849				1	
	1–10	83				1.502 (0.708, 3.186)	
	11–20	44				2.530 (1.054, 6.071)*	
	>20	149				0.940 (0.455, 1.940)	
Alcohol consumption	None	1891				1	
	Light	144				0.709 (0.364, 1.383)	
	Heavy	83				0.717 (0.298, 1.725)	
TPOAb	Negative	1686	1		1		1
	Positive	351	2.582 (1.274, 5.233)**		2.124 (0.879, 5.130)		2.20 (1.454, 3.327)**
TGAb	Negative	1640	1		1		1
	Positive	397	2.373 (1.171, 4.812)*		2.289 (0.952, 5.501)		1.398 (0.935, 2.091)
Thyroid nodules	No	1753	1	1			1
	Yes	329	4.383 (2.455, 7.826)**	0.492 (0.252, 0.960)*			1.769 (1.222, 2.561)**
Goiter	No	2025				1	1
	Yes	57				3.477 (1.997, 6.055)**	1.918 (0.963, 3.818)

*p<0.05.

**p<0.01.

#### Goiter

No significant difference was found in the prevalence of goiter among the three groups (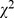 = 1.871, *p* = 0.392). Logistic-regression analysis indicated that female, TPOAb positive, TGAb positive and having nodules were risk factors for developing goiter (Table 5).

#### Hypothyroidism

The prevalence of overt hypothyroidism was 2.56%, 1.18% and 1.05% for the iodine excess, sufficient and deficient groups, respectively. A significant difference was found among the three groups (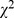 = 6.215, *p* = 0.045). However, there were no significant differences in the pairwise comparisons (Iodine excess group vs. Iodine sufficient group, 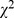 = 3.027, *p* = 0.082; Iodine excess group vs. Iodine deficient group, 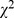 = 4.576, *p* = 0.032>0.0125; Iodine sufficient group vs. Iodine deficient group, 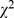 = 0.044, *p* = 0.834). No significant difference was found among the three groups in males or females (males, *p* = 0.253; females, *p* = 0.183) as shown in Table 6.

**Table 6:** Prevalence of thyroid disease within the different iodine nutrition groups.

Thyroid disease	Iodine excess group number of cases (percent)			Iodine sufficient group number of cases (percent)			Iodine deficient group number of cases (percent)		
	Total (n = 861)	Male (n = 256)	Female (n = 605)	Total (n = 509)	Male (n = 197)	Female (n = 312)	Total (n = 667)	Male (n = 157)	Female (n = 510)
Subclinical hypothyroidism	173 (20.09)	39 (15.23)	134 (22.15)	53 (10.41)	12 (6.09)	41 (13.14)	15 (2.25)	3 (1.91)	12 (2.35)
Overt hypothyroidism	22 (2.56)	6 (2.34)	16 (2.64)	6 (1.18)	1 (0.51)	5 (1.60)	7 (1.05)	1 (0.64)	6 (1.18)
Subclinical hyperthyroidism and overt hyperthyroidism	10 (1.16)	1 (0.39)	9 (1.49)	5 (0.98)	0	5 (1.60)	23 (3.44)	1 (0.64)	22 (4.31)
Subclinical hyperthyroidism	5 (0.58)	1 (0.39)	4 (0.66)	1 (0.20)	0	1 (0.32)	13 (1.95)	0	13 (2.55)
Overt hyperthyroidism	5 (0.58)	0	5 (0.83)	4 (0.79)	0	4 (1.28)	10 (1.50)	1 (0.64)	9 (1.76)
Graves' disease	3 (0.35)	0	3 (0.50)	3 (0.59)	0	3 (0.96)	5 (0.75)	1 (0.64)	4 (0.78)
Autoimmune thyroiditis	70 (8.13)	23 (8.98)	47 (7.77)	31 (6.09)	8 (4.06)	23 (7.37)	17 (2.55)	4 (2.55)	13 (2.55)
Hashimoto's thyroiditis	9 (1.05)	2 (0.78)	7 (1.16)	2 (0.39)	0	2 (0.64)	0	0	0
Atrophic thyroiditis	61 (7.08)	21 (8.20)	40 (6.61)	29 (5.70)	8 (4.06)	21 (6.73)	17 (2.55)	4 (2.55)	13 (2.55)

The prevalence of subclinical hypothyroidism was 20.09%, 10.41%, and 2.25% for the iodine excess, sufficient and deficient iodine groups, respectively. There were significant differences in the prevalence of subclinical hypothyroidism among the comparisons in males or females. In males, the prevalence of subclinical hypothyroidism differed significantly among all three groups (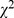 = 24.159, *p* = 0), between the iodine excess group and iodine sufficient group (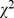 = 9.315, *p* = 0.002), and between the iodine excess group and iodine deficient group (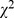 = 18.911, *p* = 0), but not between the iodine sufficient group and iodine deficient group (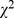 = 3.763, *p* = 0.052). In females, there were significant differences among the three groups (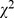 = 95.232, *p* = 0), between the iodine excess group and iodine sufficient group (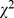 = 10.816, *p* = 0.001), and between the iodine sufficient group and iodine deficient group (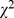 = 37.349, *p* = 0), and between the iodine excess group and iodine deficient group (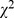 = 95.295, *p* = 0). Logistic-regression results indicated that females were more susceptible to subclinical hypothyroidism than males, and people living in the excess and sufficient areas were more susceptible to subclinical hypothyroidism than those in the deficient areas. Thyroid nodules and TPOAb positive were risk factors for subclinical hypothyroidism (Table 5).

#### Hyperthyroidism

There were no significant differences in the prevalence of overt hyperthyroidism (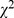 = 3.590, *p* = 0.166) and Graves' disease (*p* = 0.593, Fisher's exact test) among the three groups. The prevalence of subclinical hyperthyroidism differed significantly among the three groups (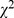 = 11.596, *p* = 0.003); significant differences were found between the iodine sufficient group and deficient group (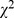 = 7.538, *p* = 0.006), but not between the iodine excess group and deficient group (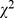 = 6.045, *p* = 0.014>0.0125).

The prevalence of subclinical hyperthyroidism and overt hyperthyroidism was 1.16%, 0.98% and 3.44% for the iodine excess, sufficient and deficient groups, respectively. The prevalence of subclinical hyperthyroidism and overt hyperthyroidism differed significantly among the three groups (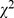 = 13.628, *p* = 0.001), between the iodine deficient group and iodine excess group (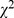 = 9.302, *p* = 0.002), and between the iodine deficient group and iodine sufficient group (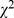 = 7.553, *p* = 0.006). Logistic-regression results indicated that female, living in an iodine deficient area, and young or middle aged were risk factors for developing overt hyperthyroidism or subclinical hyperthyroidism.

#### Autoimmune Thyroiditis

The prevalence of autoimmune thyroiditis differed significantly among the three groups (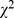 = 21.564, *p* = 0), between the iodine excess group and iodine deficient group (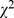 = 21.805, *p* = 0), and between the iodine sufficient group and iodine deficient group (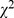 = 9.249, *p* = 0.002). The prevalence of atrophic thyroiditis differed among the three groups (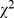 = 15.808, *p* = 0), between the iodine sufficient group and iodine deficient group (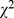 = 7.615, *p* = 0.006), and between the iodine excess group and iodine deficient group (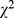 = 15.964, *p* = 0), but not between the iodine excess group and iodine sufficient group (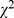 = 1.003, *p* = 0.317) (Table 6).

### TSH Level

**Figure 2:**
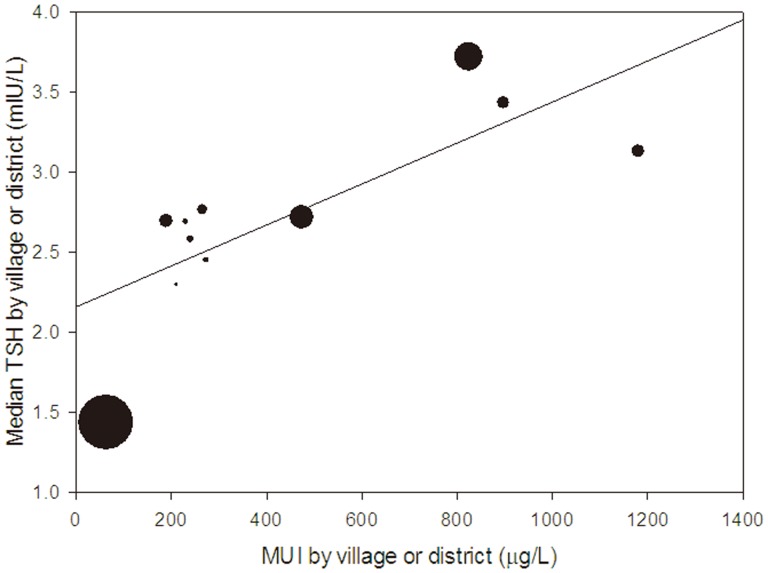
Bubble plot (clustered by village or district) of median TSH vs. MUI with a linear trend line. The size of the bubbles reflects the sample size for each village or district.

The results of the Kruskal-Wallis test indicated that the TSH levels differed significantly among the three groups (among all the three groups, *p* = 0; Iodine excess group vs. Iodine sufficient group, *p* = 0; Iodine excess group vs. Iodine deficient group, *p* = 0; Iodine sufficient group vs. Iodine deficient group, *p* = 0). Figure 2 showed the correlation between the MUI and median TSH, with the increasing MUI correlating with the increasing median TSH. By spearman correlation analysis, the urinary iodine concentration was positively related with the TSH value (r = 0.414, *p* = 0). By multiple linear regression analysis, after adjusting for gender and age, the TSH value was significantly correlated with excess iodine intake (*β* = 1.764,*P* = 0.001), iodine deficient intake (*β* = −1.219, *P* = 0.028) and TGAb positivity (*β* = 3.038, *P* = 0) (Table 7).

**Table 7:** Simple and multivariate linear regression analyses of TSH on determinants.

Variables	*β*	95% CI	*P*
**Simple linear regression**			
Age	0.038	0.007–0.068	0.016
Gender	0.371	−0.526–1.268	0.471
Smoking	0.353	−0.027–0.733	0.069
Drinking	−0.394	−1.308–0.52	0.398
Iodine status	1.574	1.101–2.047	0
TPOAb positive	3.041	1.96–4.121	0
TGAb positive	3.815	2.791–4.839	0
**Multivariate linear regression**			
Age	0.023	−0.008–0.054	0.14
Gender	0.484	−0.415–1.384	0.291
TGAb positive	3.038	1.787–4.288	0
TPOAb positive	1.307	−0.001–2.616	0.050
Excess group★	1.764	0.746–2.781	0.001
Deficient group★	−1.219	−2.307– −0.131	0.028

★ Dump variable: the reference is the sufficient group.

## Discussion

Interestingly, our findings indicated that thyroid nodules were likely to be prevalent when the population has excess or deficient iodine intake. The correlation between iodine deficiency and thyroid nodules has been reported in many countries [26], [27], [28], [29]. Takahashi et al. found that moderate iodine deficiency might be responsible for the increase of thyroid nodules in the Marshallese population [27]. The study performed by Gutekunst et al. concluded that moderate dietary iodine deficiency might cause both goiters and thyroid nodules [28]. However, research on the relationship between excess iodine intake and thyroid nodules is scarce. Recently, a cross-sectional study on the relationship between non-iodized salt and thyroid nodules conducted in Hangzhou (a foreland city in China) found a significant correlation between low urine iodine concentration and high thyroid nodules prevalence. However, in contrast to our findings, the Hangzhou study did not find a significant relationship between high and excess iodine intake and thyroid nodules [30]. In that study, the excessive iodine intake population selection didn't depend on the MUI of the population, but depended on the individual urine iodine concentration [30]. In fact, Hangzhou is another environmental iodine deficient area [31]. Future study is needed to investigate the relationship between excess iodine intake and the prevalence of thyroid nodules. Our study demonstrated that smoking 11–20 cigarettes per day was significantly related with the presence of thyroid nodules. The main reason for this might be the sample size of the 11–20 cigarettes per day group, which was much smaller than the other groups.

The results indicated that subclinical hypothyroidism was more likely to be prevalent in high iodine intake areas than in deficient or sufficient areas, while overt hyperthyroidism and subclinical hyperthyroidism were more likely to be prevalent in iodine deficient areas than in excess or sufficient areas. Likewise, our study results are similar to Andersen's study [32], in which they found more hypothyroidism and less hyperthyroidism in elderly people (aged 75–80) with sufficient iodine nutrition compared to those with mild iodine deficiency. A study conducted in China found that children had a higher concentration of TSH and a higher prevalence of thyroid diseases in the excess iodine area than in the iodine adequate area, especially for subclinical hypothyroidism [16].

It's well documented in previous studies that the prevalence of goiter in school children is higher in iodine excess or deficient areas than in iodine sufficient areas [4], [33], [34], [35], [36]; the prevalence of goiter in school-age children is usually used as a major indicator of IDD surveillance [37]. The results of this study, which included both diffuse and nodular goiter, indicated that goiter prevalence was low (<5%) in all three groups, regardless of iodine excess or deficiency. In addition, females were more susceptible to goiter. In Teng's study, the prevalence of goiter in adults in the more than adequate iodine area was higher than that in the excess iodine areas [25]. The goiter prevalence was different between adults and school children. Based on the above evidence, it could be concluded that thyroid goiter might not be an accurate indicator for assessing the iodine status in adults.

The results of this study indicated that iodine nutrition status play an important role in the TSH level, and iodine excess or deficiency influenced the TSH level. It is reported that neonatal blood TSH concentrations are an indicator for monitoring iodine deficiency in many countries [38], [39], [40]. Based on our survey, except for MUI, median TSH could be deemed as an alternative indicator for monitoring the iodine nutrition status of the adult population in iodine excess or deficient areas. Our results suggested that autoimmune thyroiditis might be prevalent in iodine excess or sufficient areas, which was in line with the previous study [25].

IDD was widespread in China until 1994 when universal salt iodization (USI) was carried out as a mandatory effective strategy to prevent IDD. Afterward, according to the IDD Surveillance Report of China, the government adjusted the iodine concentration in iodized salt three times to maintain the iodine nutrition at an appropriate level. However, at present, there are extra environmental iodine deficient areas with low iodized salt coverage in China and iodine deficiency remains a public health problem. The results of the present study indicate that Tieshangang District of Beihai City is a mild iodine deficient area with a high prevalence of thyroid nodules, overt hyperthyroidism and subclinical hyperthyroidism. The coverage rates of iodized salt and qualified iodized salt are low in Tieshangang District, and the extra environmental iodine nutrition is deficient, such as drinking water. In the coastal areas, there are illegal private salt plants that produce coarse salt (non-iodized salt), the price of course salt is lower than that of iodized salt, and thus it is easier to obtain for the households living in the coastal areas. This situation in the coastal areas highlights the necessity of consolidating the USI and ensuring a high coverage rate of qualified iodized salt, which are indispensable for preventing IDD, especially in coastal areas in China. Adequate iodine nutrition protects the health of the thyroid and prevents a high prevalence of thyroid disease. There are 30 million people living in the high iodine water areas distributed in 734 towns in China [13]. One of the effective measures to prevent excess iodine intake is to sell non-iodized salt in the market in high iodine water areas; the water authorities seldom pay attention to the iodine concentration in the water improvement program. This study demonstrated that the current intervention of supplying non-iodized salt was not enough to prevent excess iodine intake in high iodine water areas; therefore, changing to a safe water source may be the most effective measure for the prevention of the harm resulted from excess iodine intake.

Currently, the subjects of regular iodine nutrition surveillance are children, pregnant women and lactating women. However, our study suggested that both excess and deficient iodine intake could cause thyroid dysfunction or disease in adults; therefore, the adult population should also be under surveillance.

The present study has some limitations. First, the proportion of gender among the three different iodine nutrition groups was different, which might result in a bias for the prevalence of the thyroid disease; however, we applied the multivariable regression analysis to exclude the potential influence of confounders. Second, our survey sites were selected from three provinces in China, the dietary habits may be different in these sites, and we did not conduct a dietary survey, which may affect the results of this study.

In conclusion, iodine deficiency and iodine excess coexist in China. We have demonstrated that both iodine excess and deficiency induce thyroid dysfunction or diseases. The high iodine water areas should decrease iodine intake by changing to a safe water source without providing non-iodized salt, whereas in coastal areas, the non-iodized salt should be forbidden and we should encourage the household to buy iodized salt. These measures should be adopted to make sure that the MUI is maintained at an optimal level (100–199 µg/L) as suggested by the WHO [37]. Our study has provided important baseline data for monitoring the iodine nutrition and thyroid function in iodine excess or deficient areas. In view of our findings, it is recommended that, in the future, except for children, pregnant women and lactating women, an increased emphasis should be placed on monitoring the iodine status of the adult population, especially in the endemic regions.
